# Proof-of-concept trial of the combination of lactitol with *Bifidobacterium bifidum* and *Lactobacillus acidophilus* for the eradication of intestinal OXA-48-producing *Enterobacteriaceae*

**DOI:** 10.1186/s13099-020-00354-9

**Published:** 2020-04-07

**Authors:** Juan Carlos Ramos-Ramos, Fernando Lázaro-Perona, José Ramón Arribas, Julio García-Rodríguez, Jesús Mingorance, Guillermo Ruiz-Carrascoso, Alberto M. Borobia, José Ramón Paño-Pardo, Rafael Herruzo, Francisco Arnalich

**Affiliations:** 1 Unidad de Microbiología Clínica y Enfermedades Infecciosas, Servicio de Medicina Interna, Hospital Universitario La Paz, Paseo de La Castellana 261, 28046 Madrid, Spain; 2 Servicio de Microbiología, Hospital Universitario La Paz, IdiPaz, Paseo de La Castellana 261, 28046 Madrid, Spain; 3 Departamento de Farmacología Clínica, Hospital Universitario La Paz, Paseo de La Catellana 261, 28046 Madrid, Spain; 4 Servicio de Medicina Preventiva, Hospital Universitario La Paz, Paseo de La Castellana 261, 28046 Madrid, Spain; 5 Servicio de Medicina Interna, Hospital Universitario La Paz, Paseo de La Castellana 261, 28046 Madrid, Spain; 6 Present Address: Division of Infectious Diseases, Hospital Clínico Universitario “Lozano Blesa”, Zaragoza, Spain; 7 Present Address: Instituto de Investigaciones Sanitarias (IIS) de Aragón, Zaragoza, Spain

**Keywords:** OXA-48-producing *Enterobacteriaceae*, Intestinal colonization, Prebiotics, Probiotics, Carbapenemases

## Abstract

**Background:**

The major reservoir of carbapenemase-producing *Enterobacteriaceae* (CPE) is the gastrointestinal tract of colonized patients. Colonization is silent and may last for months, but the risk of infection by CPE in colonized patients is significant.

**Methods:**

Eight long-term intestinal carriers of OXA-48-producing *Enterobacteriaceae* (OXA-PE) were treated during 3 weeks with daily oral lactitol (Emportal^®^), *Bifidobacterium bifidum* and *Lactobacillus acidophilus* (Infloran^®^). Weekly stool samples were collected during the treatment period and 6 weeks later. The presence of OXA-PE was investigated by microbiological cultures and qPCR.

**Results:**

At the end of treatment (EoT, secondary endpoint 1), four of the subjects had negative OXA-PE cultures. Three weeks later (secondary endpoint 2), six subjects were negative. Six weeks after the EoT (primary endpoint), three subjects had negative OXA-PE cultures. The relative intestinal load of OXA-PE decreased in all the patients during treatment.

**Conclusions:**

The combination of prebiotics and probiotics was well tolerated. A rapid reduction on the OXA-PE intestinal loads was observed. At the EoT, decolonization was achieved in three patients.

*Clinical Trials Registration:* NCT02307383. EudraCT Number: 2014-000449-65.

## Introduction

OXA-48-producing *Enterobacteriaceae* (OXA-PE) are part of the global epidemic of carbapenemase-producing *Enterobacteriaceae* (CPE), a problem that has spread to many hospitals around the world. In December 2010, an outbreak of OXA-48-producing *Klebsiella pneumoniae* was identified in our hospital, and since then we have faced an endemic situation that has involved hundreds of patients [1].

The major reservoir of CPE is the gastrointestinal tract of the colonized patients. This might complicate the control of outbreaks since colonization may be silent and may last for months [2, 3]. The identification and isolation of colonized patients is one of the key strategies for the control of the CPE transmission.

One supportive measure for the control of colonization is selective intestinal decontamination of CPE by oral non-absorbable antibiotics active against aerobic gram-negative rods (generally colistin and aminoglycosides). This measure is used as a prophylaxis to prevent intestinal translocations in neutropenic patients, and also for prevention of pneumonia associated with mechanical ventilation in intensive care units. Using these antibiotics might be accompanied by a certain risk of promoting antibiotic resistance, but several studies have found it to be non-significant [4, 5].

Another strategy recently proposed for the control of colonization by CPE is the use of probiotics to displace them. Probiotics are live microorganisms (*Lactobacillus acidophilus, Bifidobacterium bifidum*) that may induce beneficial changes in the gut microbiome and modulate the immunologic status of the patient [6]. Probiotics may be co-administered with prebiotics, non-absorbable compounds (such as lactulose and lactitol) that are metabolized by the gut microbiota and selectively favor the proliferation of microorganisms such as *Bifidobacterium* spp. and *Lactobacillus* spp. Lactitol acts through decreasing the intestinal pH to favor the growth of acidophilic microbiota while inhibiting the growth of *Enterobacteriaceae* [7].

Some studies have reported beneficial effects of using probiotics for the eradication of pathogenic bacteria [8], though others found no significant effects [9–11]. No studies exist regarding the use of probiotics for the decolonization of CPE in chronic and long-term carriers. Therefore, the objective of this work was to evaluate the safety and efficacy of the co-administration of prebiotics (lactitol) and probiotics (*Lactobacillus acidophilus* and *Bifidobacterium bifidum*) in reducing the intestinal colonization of OXA-PE in long-term carriers with normal nutritional and immunological statuses.

## Results

### Study design

We designed a single arm, open label, pilot clinical trial with long-term carriers of OXA-PE to evaluate the efficacy of the oral administration of probiotics (Infloran^®^, *Bifidobacterium bifidum* and *Lactobacillus acidophilus,* 2 × 10^9^ CFU tid–po) and prebiotics (Emportal^®^, lactitol 10 g tid) during 3 weeks, in order to evaluate intestinal decolonization. The trial was registered with EudraCT (Number: 2014-000449-65) and ClinicalTrials.gov (Identifier: NCT02307383). Inclusion criteria were subjects between 18 and 75 years old that had been colonized by OXA-PE during a previous hospitalization, continued being colonized for more than 6 months and had a positive screening of OXA-PE upon recruitment (exclusion criteria are detailed in section “Methods”).

The hospital records for 918 patients that had been colonized by OXA-48-producing *K. pneumoniae* in the period between 2010 and 2014 were reviewed and 22 subjects were found to meet all the inclusion and exclusion criteria. Eight of them had a positive initial screening and agreed to participate in the study.

**Fig. 1:**
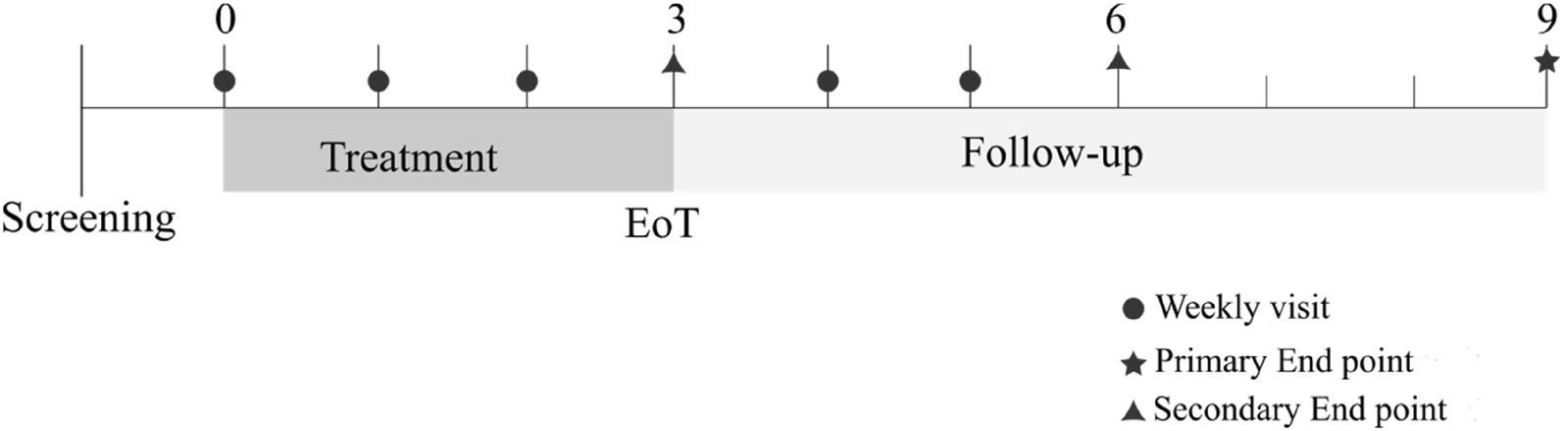
Timeline of treatment and sample collection during the study. The numbers represent weeks since the baseline visit (week 0). EoT: End of treatment. Primary endpoint: sustained gastrointestinal eradication of OXA-PE at week 6 after the EoT. Secondary endpoints: decolonization of OXA-PE at the EoT and 3 weeks after the EoT

The subjects were given the treatment for 3 weeks, and monitoring was performed through weekly visits during the treatment period, and continued for another 6 weeks afterwards (Fig. 1). During these visits, side effects were monitored and the stool samples analyzed in this study were taken. No adverse events were reported, including hepatic, renal, blood count and electrolytic disturbances. The tolerability of the treatment was good, and the only side effects related with the use of lactitol were flatulency in 4/8 (50%) of the subjects and mild diarrhea that disappeared spontaneously without stopping lactitol in 1/8 (12.5%) of the subjects. All side effect symptoms disappeared after the withdrawal of lactitol.

### Treatment outcomes

Six weeks after the EoT (week 9, primary endpoint) three subjects (37.5%) had negative cultures of OXA-PE, with two of them having had negative cultures in the last three visits of the study (weeks 5, 6 and 9). Regarding the secondary end points, four of the subjects (50%) had negative cultures for OXA-PE at the EoT (week 3), and that number increased to six (75%) 3 weeks after the EoT (week 6).

Overall, six of the subjects presented intermittent negative cultures during the whole study. In one of the subjects the cultures for OXA-PE were positive in all the samples, and in another subject no OXA-PE was recovered throughout the study.

### Monitoring of the relative load of OXA-PE during treatment

**Fig. 2:**
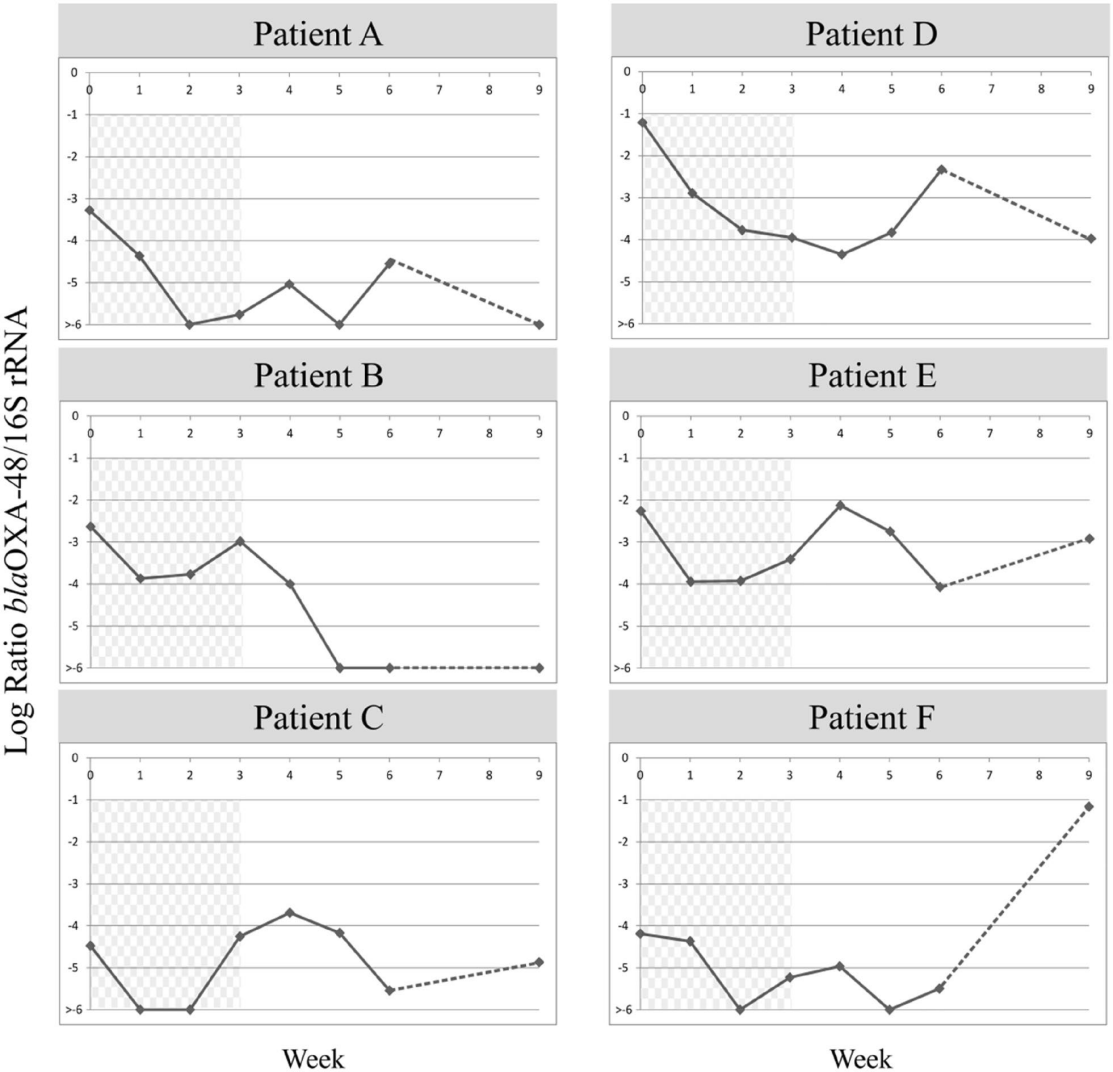
Evolution of the relative intestinal load of OXA-PE during the study. The horizontal axis shows weeks since the beginning of treatment, starting at the baseline visit. The darkened area highlights the treatment period. The discontinuous line represents the lapse of time between the samples obtained at the third and sixth weeks after EoT

The relative abundance of OXA-PE could be determined in six subjects. The remaining two had negative qPCR for *bla*_OXA-48_ in all the samples, despite having positive cultures. In the six subjects with positive qPCR results, the baseline logarithm of the fraction of OXA-PE relative to the total fecal bacteria ranged from − 1.21 to − 4.47, which is 6.15% and 0.003% of the total bacterial population, respectively (Fig. 2). After 2 weeks of oral treatment, the OXA-PE fraction showed a reduction of more than one logarithm in all cases. The relative loads for the three subjects that had a baseline load of OXA-PE below − 3 reached undetectable levels, while the reduction ranged between 1.13 and 2.55 logarithms in the other three subjects. In all of these subjects, the samples obtained after the end of treatment showed transitory increases of the relative OXA-PE loads, but the relative loads at the end of the study were varied: in two subjects, OXA-PE was no longer detectable by qPCR, one subject had a reduction of three logarithms, two subjects almost recovered the baseline levels, and the last subject had an increase in the relative abundance of OXA-PE (Fig. 2).

## Discussion

In this study, we have tested the efficacy of 3 weeks of oral treatment with a combination of lactitol and probiotics (*Bifidobacterium bifidum* and *Lactobacillus acidophilus*) for intestinal decolonization of OXA-PE in eight healthy volunteers that were long-term carriers. The period of treatment was set for 3 weeks based on previous experience that suggested a minimum of 1 week of treatment in order to be able to evaluate its efficacy. Safety and tolerance throughout the study’s period were good and treatment costs were moderate (on the order of 50€). Apparent decolonization of OXA-PE six weeks after the EoT (week 9, primary endpoint) was achieved in three subjects (37.5%). The relative intestinal load of OXA-PE consistently decreased in all six tested subjects during treatment, in three of them below detectable levels. The fact that in all of them increased again after the EoT shows that colonization may persist with loads below the limits of detection and suggests that longer treatments might be needed to effectively reach decolonization. Six weeks after the EoT, the OXA-PE loads in five out of the six subjects were reduced with respect to their initial values. The decolonization rate obtained in our study, without using antibiotics, was similar to the values reported at 1 or 3 months of treatment in several studies with non-absorbable antibiotic decolonization regimes (25%) [12]. Similar results have been obtained with fecal microbiome transplantation (FMT) without antibiotics [13–15], though the FMT protocols need further testing and standardization for CPE eradication [16, 17].

It has been shown that CPE are spontaneously cleared from the intestine after some time [18], and indeed, 14 out of the 22 candidates that met the inclusion and exclusion criteria of our study had already become negative before the onset of the study. The time since discharge was longer in these 14 subjects than in the 8 positive subjects [3], suggesting that spontaneous clearance would eventually occur in these subjects as well. In the positive subjects, the original hospital-acquired strains have been lost, and the OXA-48 plasmid was maintained in endogenous, non-multi-resistant strains of *K. pneumoniae*, *Klebsiella oxytoca* or *Escherichia coli* [3]. The selection of long-term carriers was intended to reduce the probability of spontaneous decolonization during the treatment period. Therefore, the drastic reduction of the OXA-PE load in all of the subjects during the first 2 weeks of treatment suggests that it was an effect of the treatment. Nevertheless, a placebo control could not be included in this study due to the small sample size of the subjects.

The importance of our results lies in the fact that higher loads of CPE are associated with increased contamination of the environment surrounding the colonized patients [19] and a higher risk of spread to other wards and patients. Therefore, decreasing the intestinal loads of OXA-PE might be an effective way to reduce the risk of cross-transmission in wards harboring colonized patients. Moreover, the decrease of the OXA-PE loads below the detection limits can also reduce the risk of developing an infections during hospitalization [20–22]. This could be useful in situations where programmed surgical procedures are to be performed and for managing critically ill patients, especially since the decrease of the OXA-PE loads after the onset of treatment was fast and maintained throughout the treatment.

One of the weaknesses of our study is the small size of the study group that is not representative of the entire population of OXA-PE carriers. This was the result of the strict exclusion criteria that were put in place in order to avoid biases related to concurrent treatments or morbidities. Nevertheless, our results show that the combined use of probiotics and prebiotics has a rapid impact on the intestinal load of OXA-PE with minimal side effects. In line with recently published EUCAST guidelines [23], this strategy should be further explored among selected hospitalized patient groups who might benefit from a decrease in the intestinal OXA-PE load.

## Conclusions

The combination of prebiotics and probiotics was well tolerated and a rapid reduction on the OXA-PE intestinal loads was observed during the treatment. At the EoT apparent decolonization was achieved in three out of eight patients.

## Methods

### Subject recruitment

Eligible subjects were adults between 18 and 75 years of age, of both genders, that had been colonized by OXA-PE during hospitalization, maintained the colonization for more than 6 months and had a positive screening of OXA-PE upon recruitment. They were recruited by a phone call and a personal interview. The exclusion criteria were: hospitalization due to acute pathologies, systemic antibiotic or glucocorticoid treatments during the previous month, diarrhea 1 month before initiating the treatment, allergy or intolerance to lactitol, lactulose or probiotics, electrolytic alterations (K^+^< 3 mEq/L, Mg^++^< 1.8 mEq/L, Ca^++^< 8 mg/dL), neutropenia (< 100 × 10^3^/µL), chemotherapy or immunosuppressive treatment, liver dysfunction (ASAT/ALAT > 5 times the upper limit, AP > 3 times the upper limit, or bilirrubin > 2 mg/dl), chronic renal failure (GFR < 30 ml/min), poorly controlled diabetes (HbA1c > 8 mmol/mol), endovascular prosthesis and severe valvulopathy [3].

The study was conducted in accordance with the Declaration of Helsinki. It was approved by the Ethics Committee of Hospital Universitario La Paz on May 8th, 2014, with code number: 4131 and EudraCT number: 2014-000449-65, and by the Spanish Drugs Agency, AEMPS, on July 7th, 2014. All subjects received written and spoken information about the study during their interviews and were informed of the opportunity to participate in the study and ability to withdraw at any time without penalties. Signed informed consent was obtained from all the study’s subjects.

### Primary and secondary endpoints

The primary endpoint was the sustained gastrointestinal (GI) eradication of OXA-PE 6 weeks after the end of treatment (EoT). The secondary endpoints were the GI decolonization of OXA-PE at the EoT and 3 weeks after the EoT.

### Study procedures

Visits were performed once a week during the first 6 weeks (3 weeks of intervention and 3 weeks of follow-up period), and a final visit was performed at week 9 (6 weeks after the end of the treatment). A rectal swab was taken for the initial screening during the first visit, and stool samples were obtained throughout the following visits. If the initial screening was positive for OXA-PE, the subject was included in the study and a second visit was planned where the treatment begun. Blood tests were performed at the initial screening and the EoT to analyze hepatic and renal functions, blood counts and electrolytic disturbances. Safety and tolerability were evaluated every week since the initiation of treatment through a medical visit and physical examination.

The efficacy of the intervention was primarily evaluated 6 weeks after the EoT (sustained response) and secondarily at the EoT and 3 weeks after the EoT. The effect of the treatment on intestinal colonization by OXA-PE was monitored during the treatment period and the three following weeks using the stool samples received during every visit.

### OXA-PE identification

Rectal swabs were inoculated on OXA-PE selective agar media (chromID™ OXA-48, bioMérieux), and tryptic soy broth containing an ertapenem disk (1 µg/ml), and incubated for 18 h at 37 °C. After incubation, the broth was plated on the same agar selective medium and incubated another 18 h at 37 °C. Stool samples were processed in the same way, with the exception that before plating, a small portion of stool samples (around 0.1 g) was suspended in 0.5 ml of 0.9% saline that was used to inoculate the media. Isolates were identified using MALDI Biotyper^®^ (Bruker Daltonics) and real time PCR (OXVIKP, Progenie Molecular^®^) was used to confirm the presence of the *bla*_OXA-48_ gene.

### Molecular methods

For DNA extraction, rectal swabs or 0.1 g of stool samples were suspended in 1 ml of saline solution (0.9% Sodium Chloride solution, Fresenius Kabi) and lysed by heating at 95 °C during 20 min. Samples were then centrifuged 1 min at 12,000 rpm to eliminate solid residues and DNA in the supernatant was extracted using the automated MagPurix^®^ system (Zinexts Life Science Corp.). For characterization of qPCR parameters, 1 ml of a 0.5 McFarland suspension of an OXA-48-producing *K. pneumoniae* isolate was extracted using the method described above. Primers and TaqMan probes targeting the *bla*_OXA-48_ gene [24] and the *16SrRNA* gene [25] were used as previously described. Both probes were designed with the FAM reporter dye.

qPCRs were carried out in two parallel assays targeting the *bla*_OXA-48_ gene and the *16SrRNA* gene. Each reaction tube contained 0.1 µM of the specific probe, 1 µM of each forward and reverse primers, 10 µl of Premix Ex Taq™ (Takara Bio Inc.), 2.8 µl of molecular biology-grade water and 5 µl of template DNA. Reactions were carried out on the CFX96 Touch™ Real-Time PCR Detection System (Bio-Rad Laboratories) with the following cycling conditions: One holding period of 10 min at 95℃ followed by 45 two-step cycles consisting of 30 s at 95℃ and 1 min at 60℃. The Threshold cycles (C_t_) were automatically calculated by the PCR system.

**Fig. 3:**
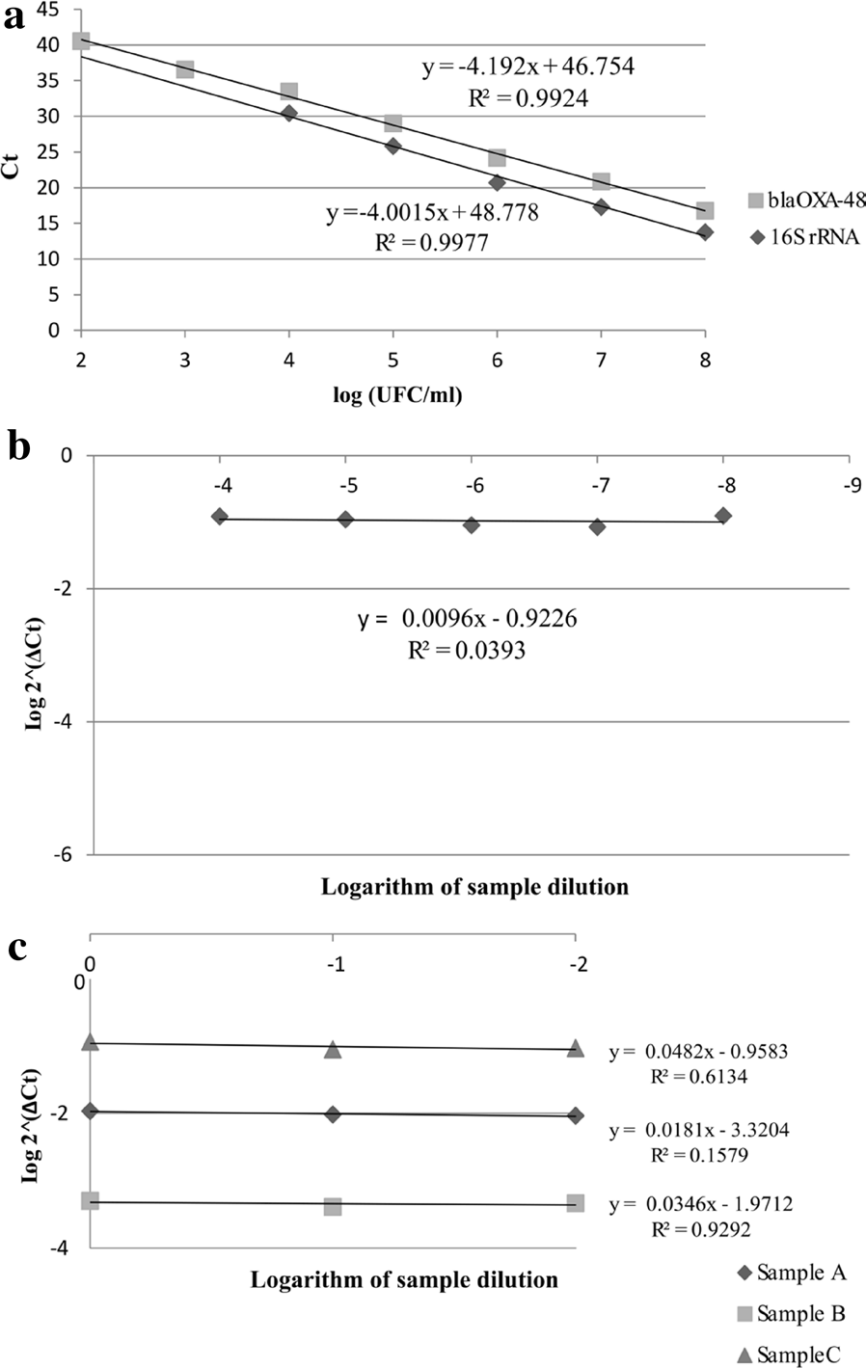
Validation of the qPCR Reactions. **a** Determination of linearity and the limit of detection for the two qPCR reactions using tenfold serial dilutions of DNA extracted from 1 ml of a 0.5 McFarland suspension of an OXA-48-producing *K. pneumoniae*. **b** The log ratio of the two qPCR reactions plotted against tenfold serial dilutions of the OXA-48-producing *K. pneumoniae*. **c** The log ratio of the two qPCR reactions plotted against tenfold serial dilutions of rectal swabs having different loads of *bla*_OXA-48_

The linear ranges and the limits of detection of qPCRs targeting both *16SrRNA* and *bla*_OXA-48_ were determined using tenfold serial dilutions of DNA extracted from 1 ml of a 0.5 McFarland suspension of an OXA-48-producing *K. pneumoniae*. For both qPCRs, we established the linear range over 1000,000-fold range dilutions and created a standard curve. Efficiency (Ef) was calculated based on the standard curve slope (Ef = 10^(−1/slope)^-1). Individual qPCR parameters were analyzed with LinRegPCR 11 [26]. The linear range of the qPCR_16S_ was maintained for five logarithmic dilutions (8-4 log CFU/ml, R^2^ = 0.992), and the linear range of qPCR_OXA-48_ for seven logarithmic dilutions (8-2 log CFU/ml, R^2^ = 0.997). Efficiencies for qPCR_16S_ and qPCR_OXA-48_ were 1.847 and 1.850, respectively. The limit of detection was set below 100 CFU/ml for both *16SrRNA* and *bla*_OXA-48_ (Fig. 3a). The effect of DNA dilution on the ΔC_t_ was calculated in the OXA-48-producing *K. pneumoniae* strain, and three rectal swabs obtained from colonized patients. Variation of the ΔC_t_ with respect to template dilution was considered insignificant when the slope of the log CFU/ml–ΔC_t_ curve is close to 0 (Fig. 3b, c). The efficiency of the qPCR was found to be independent from the sample or the DNA dilution. All reactions were carried out by duplicate for stool samples or triplicate for the standard curves and qPCR testing.

DNA from stool samples was diluted tenfold in order to avoid PCR inhibition. When no detection of the *bla*_OXA-48_ gene was possible, non-diluted DNA was used, and 100-fold dilutions were used in case of PCR inhibition or when the C_t_ values were out of the linearity range. Intestinal loads were calculated from the difference between the C_t_ of the qPCR targeting the *bla*_OXA-48_ and the C_t_ of the reference gene, the *16SrRNA*, using the 2^−ΔCt^ method [27, 28]. In order to use the method, several requirements had to be met. Mainly, the efficiencies of the two qPCRs had to be similar and > 80%, and the dilution of the DNA template should not influence the ΔC_t_ results. These conditions were met in our validation experiments described above. Moreover, the ΔC_t_ method assumes a one-to-one ratio of *bla*_OXA-48_ to *16SrRNA* genes per cell. We detected an average of three copies per cell of the *bla*_OXA-48_ gene in *K. pneumoniae*, and have calculated that an average of four copies of the *16SrRNA* per cell across the organisms that typically populate the intestinal microbiome (median value) using the *rrn*DB database (https://rrndb.umms.med.umich.edu/).

## Data Availability

The datasets generated and/or analysed during the current study are available from the corresponding author upon reasonable request.
